# Circulating CCR6 ^+^ ILC proportions are lower in multiple sclerosis patients

**DOI:** 10.1002/cti2.1426

**Published:** 2022-12-23

**Authors:** Florentina Aglas‐Leitner, Pierre Juillard, Anette Juillard, Scott N Byrne, Simon Hawke, Georges E Grau, Felix Marsh‐Wakefield

**Affiliations:** ^1^ Vascular Immunology Unit, School of Medical Sciences, Faculty of Medicine and Health The University of Sydney Sydney NSW Australia; ^2^ Medical University of Vienna Vienna Austria; ^3^ Centre for Immunology and Allergy Research The Westmead Institute for Medical Research Sydney NSW Australia; ^4^ Faculty of Medicine and Health, School of Medical Sciences The University of Sydney Sydney NSW Australia; ^5^ Central West Neurology and Neurosurgery Orange NSW Australia; ^6^ Liver Injury & Cancer Program Centenary Institute Sydney NSW Australia; ^7^ Human Cancer & Viral Immunology Laboratory The University of Sydney Sydney NSW Australia

**Keywords:** alemtuzumab, CCR6, ILC, MS, NK cells

## Abstract

**Objectives:**

The role of innate lymphoid cells (ILC), particularly helper ILC, in the pathogenesis of multiple sclerosis (MS) is not well understood. Here, we present a comprehensive analysis of peripheral ILC subsets in MS patients prior and after alemtuzumab administration using mass cytometry.

**Methods:**

Circulating ILC were analysed by mass cytometry in MS patients before and after alemtuzumab. These were compared with non‐MS controls. MS‐related shifts among ILC immunophenotypes were further elucidated by fast interpolation‐based t‐SNE (Flt‐SNE) dimensionality reduction.

**Results:**

Neither natural killer (NK) cells nor helper ILC (ILC1, ILC2 and ILC3) levels were altered following alemtuzumab treatment. However, CD56^bright^ NK cell expansions were observed in relapsing patients. MS patients prior to alemtuzumab further displayed proportional shifts from ILC1 to ILC2, with MS‐associated decreases in CCR6^+^ helper ILC proportions.

**Conclusion:**

CD56^bright^ NK cells during relapse indicate an immediate response to disease reactivation, while CCR6‐related shifts among helper ILC suggest altered ILC migration to the CNS during MS.

## Graphical abstract

In this study, we found CD56^bright^ natural killer (NK) cells increased in relapsing multiple sclerosis (MS) patients. MS patients further demonstrated proportional shifts in type 1 innate lymphoid cells (ILC1), ILC2 and CCR6^+^ ILC compared with non‐MS controls. Our study elucidates MS‐ and alemtuzumab‐induced dynamics within the circulating ILC population.

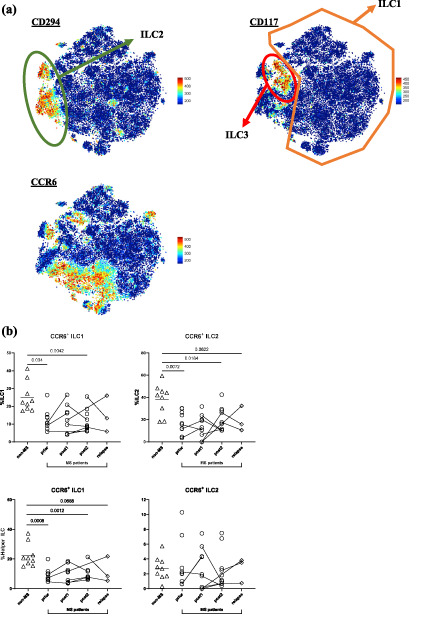

## Introduction

Alemtuzumab (*Lemtrada*™) is a disease‐modifying therapy (DMT) for multiple sclerosis (MS) treatment.^1^ As a humanised monoclonal IgG1‐antibody, it targets the surface molecule CD52 and consequently leads to the elimination of CD52‐expressing cells.^1^ Following the rapid depletion of CD52^+^ immune cells, a more tolerogenic immune cell compartment is thought to develop,^2^ though systemic autoimmunity can still be induced.^3^ To date, exact mechanisms by which transient depletion of lymphoid populations by alemtuzumab induces long‐term immune tolerance to the central nervous system (CNS) in MS are not known.^2^ Within circulation, B and T cells are primarily eradicated,^4^ which are thought to be pivotal effectors of MS pathology.^5^ However, more recently innate immune cells are considered as increasingly important in MS pathogenesis.^6^ Concomitantly, various innate immune cell populations have also been reported to express low levels of CD52,^1^, ^3^ including innate lymphoid cells (ILC).^3^

Innate lymphoid cells have lymphoid morphology but do not express adaptive lymphocyte antigen‐specific receptors.^7^, ^8^ They are categorised into different subgroups, which closely match different T cell subsets based on their cytokine and transcriptional profile and include natural killer cells (CD56^bright^ and CD56^dim^ NK cells), lymphoid tissue inducer cells (LTi) and helper ILC1, ILC2 and ILC3^7^, ^9^ (which are then further divided into NCR^+^ ILC3 and CCR6^+^ LTi‐like ILC3^10^).

Disproportionate ILC are implicated in numerous autoimmune diseases, such as type I diabetes mellitus, rheumatoid arthritis, systemic lupus erythematosus, systemic sclerosis, psoriasis and spondylarthritis.^11^, ^12^ In MS, increased circulatory pro‐inflammatory helper ILC1 and ILC3 have been described in untreated MS patients,^13^, ^14^ as well as increased ILC3 in CSF during early stages of MS.^15^ By contrast, NK cells, in particular the CD56^bright^ subpopulation, have been suggested as immunoregulatory, with observed increases under various disease‐modifying treatments (DMTs).^16^

Here, we present the results of a comprehensive analysis of the entire ILC compartment in peripheral blood mononuclear cells (PBMC) in MS patients treated or untreated with alemtuzumab. We demonstrate relapse‐related expansions in CD56^bright^ NK cells. Furthermore, CCR6‐associated ILC1 and ILC2 shifts within the helper ILC compartment were associated with MS but did not change under alemtuzumab treatment. CCR6^+^ immune cells, such as Th17 cells^17^ and more recently also CCR6^+^ ILC3,^18^ demonstrate CNS migration in experimental autoimmune encephalomyelitis (EAE),^17^, ^18^ such that the CCR6‐CCL20 axis has been proposed as a future therapeutic target.^17^ Hence, future investigations should especially regard CCR6 expression in ILC for its role in ILC migration to the CNS and CSF in MS.

## Results

To investigate the potential long‐term effects of alemtuzumab on ILC compartments in MS patients, we designed a 43‐parameter CyTOF panel (Supplementary table 1) to identify different ILC subtypes in PBMC derived from relapse‐remitting MS (RRMS) patients (see Supplementary tables 2 and 3 for patient description) before (*prior*), at various timepoints after alemtuzumab treatment (*post1* < 12 months, *post2*  > 12 months), and in patients who relapsed following alemtuzumab treatment (*relapse*).

**Figure 1:**
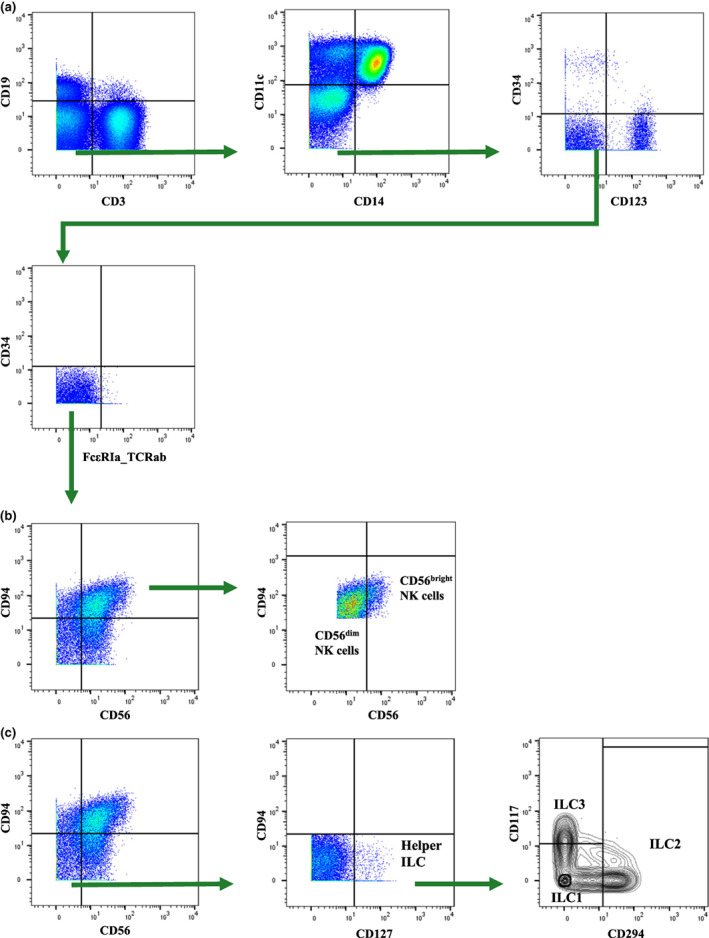
Gating strategy for ILC. **(a)** Biaxial gating for Lin^−^ (CD3^−^CD19^−^CD14^−^CD11c^−^CD123^−^CD34^−^FcεRIα^−^TCRab^−^) live CD45^+^ PBMC (peripheral blood mononuclear cells). **(b)** Natural killer (NK) cells were defined as Lin^−^CD56^+^CD94^+^ and subdivided into CD56^dim^ and CD56^bright^ NK cells. **(c)** Helper ILC were defined as Lin^−^CD56^−^CD94^−^CD127^+^ and subdivided into innate lymphoid cell (ILC) type 1 (CD294^−^CD117^−^), ILC2 (CD294^+^CD117^+/−^) and ILC3 (CD294^−^CD117^+^).

Innate lymphoid cells were defined as CD45^+^ PBMC, negative for lineage markers (Lin^−^) CD3, CD19, CD14, CD11c, CD123, CD34, FcεRIα and TCRab (Figure 1a). Lin^−^ cells were gated into Lin^−^CD56^+^CD94^+^ NK cells (and then further subdivided into CD56^bright^ and CD56^dim^ NK cells) (Figure 1b), and Lin^−^CD56^−^CD94^−^CD127^+^ helper ILC (referred to as Lin^−^CD127^+^ helper ILC), which were then subdivided into (CD294^−^CD117^−^ ILC1, CD294^+^CD117^+/−^ ILC2 and CD294^−^CD117^+^ ILC3) (Figure 1c).

**Figure 2:**
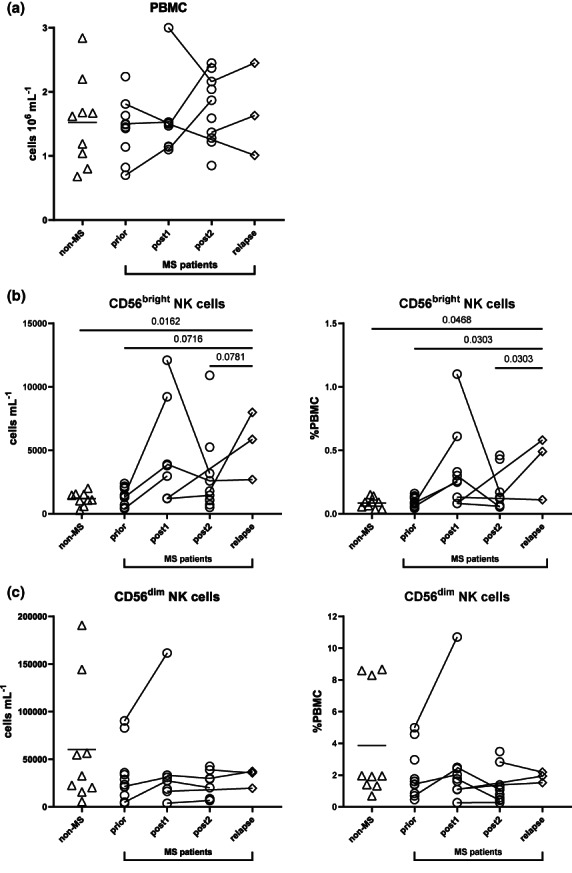
Alterations in CD56^bright^ and CD56^dim^ NK cells follow relapses but are not a long‐term effect of alemtuzumab treatment. **(a)** Peripheral blood mononuclear cell (PBMC) counts (10^6^ cells mL^−1^) across patients. Cell counts (cells mL^−1^) and percentages (as proportion of PBMC) of **(b)** CD56^bright^ NK cells and **(c)** CD56^dim^ NK cells. NK cells were defined as live CD45^+^Lin^−^ (CD3^−^CD19^−^CD14^−^CD11c^−^CD123^−^CD34^−^FcεRIα^−^TCRab^−^) CD56^+^CD94^+^. For comparisons of cell counts and percentages between all five groups (*non‐MS* (multiple sclerosis) controls (*n* = 9), untreated MS patients (*prior*, *n* = 11) and MS patients *post1* (< 12 months after alemtuzumab dose, *n* = 8/9 for cells mL^−1^ and *n* = 9/9 for proportion of PBMC), *post2* (> 12 months, *n* = 10) alemtuzumab and *relapse* (*n* = 3)), a PERMANOVA was done followed by pairwise comparisons with Holm's correction. *Prior*, *post2* and *relapse* groups were compared with *non‐MS* controls (for three comparisons). A linear mixed‐effects model was calculated when comparing between MS patients before and after treatment. 4999 permutations were then run to calculate *P*‐values. Five multiple comparisons were made (*prior* to *post1*, *post2* and *relapse*, and *post1* to *post2*, and *post2* to *relapse*) using a further 4999 permutations with Holm's correction. Mean is shown in *non‐MS* controls, *P*‐values < 0.1 are shown.

Firstly, no significant changes in PBMC counts were reported between groups (Figure 2a). There were no differences in NK cell subset levels between *non‐MS*, *prior*, *post1* and *post2* (Figure 2b and c). During our study, three patients relapsed after 34, 39 and 41 months following their first course of alemtuzumab. In these three participants, CD56^dim^ NK cells remained stable during relapse (Figure 2c). However, although only three study participants relapsed, CD56^bright^ NK cells were markedly increased in counts and proportions in this patient cohort when compared to *non‐MS* (Figure 2b, *P* = 0.0162 (cells mL^−1^); *P* = 0.0468 (%PBMC)), or *prior* (Figure 2b, *P* = 0.0716 (cells mL^−1^); *P* = 0.0303 (%PBMC)) and *post2* (Figure 2b, *P* = 0.0781 (cells mL^−1^); *P* = 0.0303 (%PBMC)).

**Figure 3:**
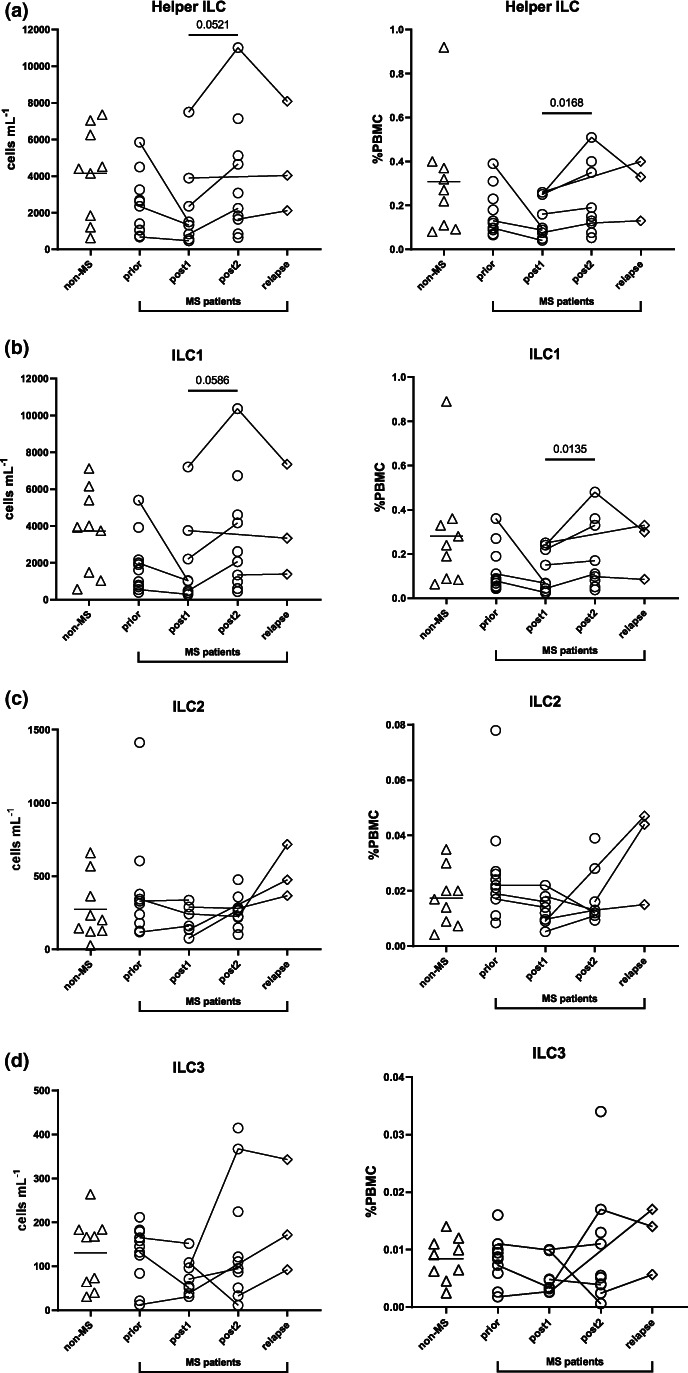
Helper ILC and subsets (cells mL^−1^ and %PBMC) following alemtuzumab. Cell counts (cells mL^−1^) and percentages (as a proportion of PBMC) of **(a)** Lin^−^CD56^−^CD94^−^CD127^+^ helper ILC and subsets **(b)** ILC1 (CD294^−^CD117^−^), **(c)** ILC2 (CD294^+^CD117^+/−^) and **(d)** ILC3 (CD294^−^CD117^+^). Helper ILC were defined as live CD45^+^Lin^−^CD127^+^ (CD3^−^CD19^−^CD14^−^CD11c^−^CD123^−^CD34^−^FcεRIα^−^TCRab^−^) CD56^−^CD94^−^. For comparisons of cell counts and percentages between all five groups (*non‐MS* (multiple sclerosis) controls (*n* = 9), untreated MS patients (*prior*, *n* = 11) and MS patients *post1* (< 12 months after alemtuzumab dose, *n* = 8/9 for cells mL^−1^ and *n* = 9/9 for proportion of PBMC), *post2* (> 12 months, *n* = 10) alemtuzumab and *relapse* (*n* = 3)), a PERMANOVA was done followed by pairwise comparisons with Holm's correction. *Prior*, *post2* and *relapse* groups were compared with *non‐MS* controls (for three comparisons). A linear mixed‐effects model was calculated when comparing between MS patients before and after treatment. 4999 permutations were then run to calculate *P*‐values. Five multiple comparisons were made (*prior* to *post1*, *post2* and *relapse*, and *post1* to *post2*, and *post2* to *relapse*) using a further 4999 permutations with Holm's correction. Mean is shown in *non‐MS* controls, *P*‐values < 0.1 are shown.

We next explored the potential long‐lasting effects of alemtuzumab on Lin^−^CD127^+^ helper ILC and their subsets (ILC1, ILC2 and ILC3) (Figure 3a–d). There were no differences in ILC2 (Figure 3c) or ILC3 (Figure 3d). There was, however, an increase in total helper ILC in both counts (Figure 3a, *P* = 0.0521) and proportions (Figure 3a, *P* = 0.0168) from *post1* to *post2*, with similar results for ILC1 (Figure 3b, *P* = 0.0586 (cells mL^−1^) and 0.0135 (%PBMC)).

**Figure 4:**
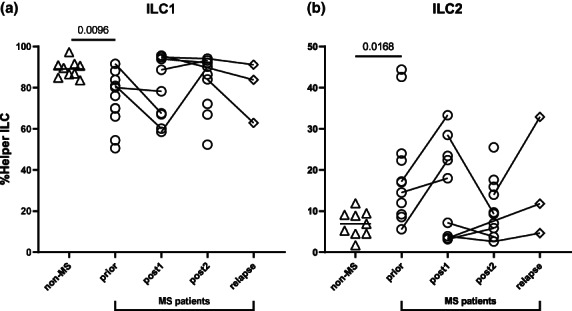
ILC subsets shift within the Lin^−^CD127^+^ helper ILC compartment in MS patients. **(a)** Levels of ILC1 (CD294^−^CD117^−^) and **(b)** ILC2 (CD294^+^CD117^+/−^) as proportions of helper ILC across timepoints (%Helper ILC). Helper ILC were defined as live CD45^+^Lin^−^CD127^+^ (CD3^−^CD19^−^CD14^−^CD11c^−^CD123^−^CD34^−^FcεRIα^−^TCRab^−^) CD56^−^CD94^−^. For comparisons of cell percentages across timelines (*non‐MS* (multiple sclerosis) controls (*n* = 9), untreated MS patients (*prior*, *n* = 11) and MS patients *post1* (< 12 months after alemtuzumab dose, *n* = 9), *post2* (> 12 months, *n* = 10) alemtuzumab and *relapse* (*n* = 3)), a PERMANOVA was done followed by pairwise comparisons with Holm's correction. *Prior*, *post2* and *relapse* groups were compared with *non‐MS* controls (for three comparisons). Five multiple comparisons were made (*prior* to *post1*, *post2* and *relapse*, and *post1* to *post2*, and *post2* to *relapse*) using a further 4999 permutations with Holm's correction. 4999 permutations were run to calculate *P*‐values. Mean is shown across groups, *P*‐values < 0.1 are shown.

Remarkably, when analysing proportions of helper ILC1, ILC2 and ILC3 within the Lin^−^CD127^+^ helper ILC compartment (as proportion of helper ILC), substantial shifts in ILC1 and ILC2 (Figure 4a), but not ILC3 (Supplementary figure 1a), were found in MS patients. In comparison with *non‐MS* controls, MS patients *prior* to alemtuzumab onset demonstrated significantly lower ILC1, but higher ILC2 proportions among helper ILC (Figure 4a, ILC1, *P* = 0.0096; ILC2, *P* = 0.0168), which were not apparent postalemtuzumab treatment (Figure 4a).

**Figure 5:**
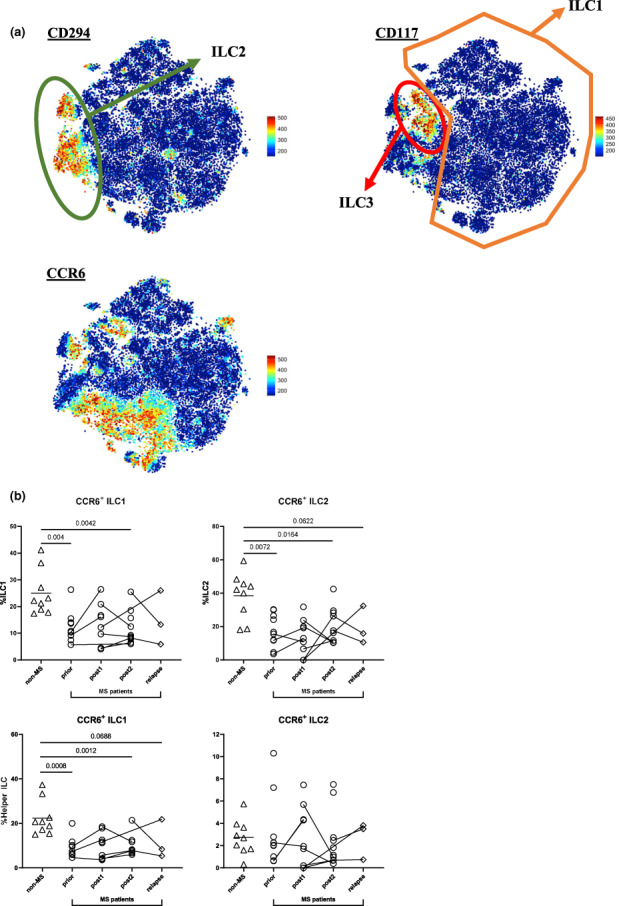
CCR6^+^ ILC1 and ILC2 are reduced in MS patients. **(a)** FIt‐SNE plots generated on helper ILC (live CD45^+^Lin^−^CD127^+^ (CD3^−^CD19^−^CD14^−^CD11c^−^CD123^−^CD34^−^FcεRIα^−^TCRab^−^CD56^−^CD94^−^)). Dimensionality reduction was done on all Lin^−^CD127^+^ helper ILC (from all patients). Dimensionality reduction plots were calculated using markers in Supplementary table 1. Expression levels of selected markers are shown, with helper ILC subsets ILC1 (CD294^−^CD117^−^), ILC2 (CD294^+^CD117^+/−^) and ILC3 (CD294^−^CD117^+^), as annotated. Identification of CCR6^+/−^ helper ILC. **(b)** For comparisons of cell percentages between all five groups (*non‐MS* controls (*n* = 9), untreated MS patients (*prior*, *n* = 10) and MS patients *post1* (< 12 months after alemtuzumab dose, *n* = 8/9), *post2* (> 12 months, *n* = 10) alemtuzumab and *relapse* (*n* = 3)), a PERMANOVA was done followed by pairwise comparisons with Holm's correction. *Prior*, *post2* and *relapse* groups were compared with *non‐MS* controls (for three comparisons). A linear mixed‐effects model was calculated when comparing between MS patients before and after treatment. 4999 permutations were then run to calculate *P*‐values. Five multiple comparisons were made (*prior* to *post1*, *post2* and *relapse*, and *post1* to *post2*, and *post2* to *relapse*) using a further 4999 permutations with Holm's correction. Mean is shown in *non‐MS* controls, *P*‐values < 0.1 are shown.

Next, we created Flt‐SNE plots of Lin^−^CD127^+^ helper ILC to delineate potential ILC immunophenotypes, which may explain the observed MS‐related shifts in ILC1 and ILC2 among helper ILC (Figure 5a). Flt‐SNE plots correctly identified distinct helper ILC subsets by CD294 (ILC2) and CD117 (ILC3) expression and revealed CD196^+^ (CCR6^+^) (Figure 5a) and relatively rare populations of CD103^+^ and HLA‐DR^+^ (Supplementary figure 2a and b) helper ILC.

In *non‐MS* controls, 26.06% of helper ILC were CCR6^+^ (25.02% CCR6^+^ ILC1, 38.49% CCR6^+^ ILC2, 23.70% CCR6^+^ ILC3). Furthermore, MS patients presented with significant decreases in CCR6^+^ ILC1 and ILC2 but not ILC3 proportions at *prior* (Figure 5b, ILC1, *P* = 0.004; ILC2, *P* = 0.0072 and Supplementary figure 3a) and *post2* (Figure 5b, ILC1, *P* = 0.0042; ILC2, *P* = 0.0164 and Supplementary figure 3a) when compared to *non‐MS* controls. Subsequent analysis of CCR6 as a percentage among Lin^−^CD127^+^ helper ILC confirmed the observed reductions in ILC1 at *prior*, *post2* and *relapse* timepoints (Figure 5b, *P* = 0.0008; 0.0012 and 0.0688, respectively), while CCR6^+^ ILC2 (Figure 5b) and ILC3 (Supplementary figure 3b) did not show any significant alterations among Lin^−^CD127^+^ helper ILC proportions.

## Conclusion

In this study, we monitored long‐term impacts of alemtuzumab on the ILC compartment in RRMS patients and elucidated its impacts on ILC subsets including CD56^bright/dim^ NK cells, ILC1, ILC2 and ILC3.

First, we interrogated potential alterations among NK cells. Most interestingly, CD56^bright^ NK cell percentages were significantly elevated in the three patients who relapsed. To our knowledge, there are no further reports of CD56^bright^ NK cell measurements within the first month of relapse. However, CD56^bright^ NK cells may likely exert protective effects during MS relapse, as CD56^bright^ NK cell expansions have been described under various potent DMT.^16^ Contrastingly, Gilmore *et al*.^19^ did not observe enhanced CD56^bright^ NK cell percentages during active disease (defined as new relapses and/or T2 lesions) in alemtuzumab‐treated patients. Possibly, the observed CD56^bright^ NK cell increment in our three relapsing MS patients is an immediate reaction to disease reactivation and hence also not displayed at baseline (*prior*), at which the vast majority of our included MS patients demonstrated MS activity (defined as MRI activity up to 6 months prior to alemtuzumab). To address distinct CD56^bright^ NK cell alterations in response to immediate versus chronic disease activity, further investigations with a larger relapsing MS patient cohort are required.

Second, no significant changes in CD56^bright^ NK cells at any other timepoint following long‐term alemtuzumab‐induced immune cell reconstitution were observed, which closely resembles results by Gross *et al*.,^3^ who have reported significantly increased CD56^bright^ NK cell numbers and/or proportions at 6‐month, but not 12‐month post‐treatment. Conversely, Gilmore *et al*. have reported augmented CD56^bright^ NK cell proportions for up to 36 months after the first alemtuzumab administration.^19^ Nevertheless, differences concerning the duration of alemtuzumab‐related effects might be because of how CD56^bright^ NK cell changes are reported, that is, as cells mL^−1^ and proportions of PBMC and ILC,^3^ or proportion of lymphocytes,^19^ as well as disparate gating strategies. While Gilmore *et al*.^19^ employed FSC/SSC channels to identify lymphocytes and defined NK cells as CD3^−^CD56^+^ cells within this population, Gross *et al*.^3^ used a lineage cocktail including antibodies against CD3, CD14, CD19 and CD20 and further excluded CD123^+^ and CD11c^+^ to identify CD56^+^ NK cells. Using mass cytometry, we were not reliant on antibody cocktails and had available channels to also filter out additional non‐ILC contaminating cells^9^ such as haematopoietic precursors (CD34^+^), or basophils and mast cells (FcεRIα^+^).

Additionally, we did not observe any differences in CD56^dim^ NK cells following alemtuzumab reconstitution, similar to results by Gross *et al.*,^3^ who also reported unaltered CD56^dim^ NK cell levels 6‐month postalemtuzumab. Interestingly, in another study^20^ Gross *et al*. found significantly decreased CD56^dim^ proportions in treatment‐naïve MS patients when compared to healthy subjects, which we did not observe in our prior treatment cohort.

The second part of our study concentrated on Lin^−^CD127^+^ helper ILC. Initially, we could not find any alemtuzumab‐induced impacts on these cells and their subsets (ILC1, ILC2 and ILC3). Conversely, others have noted increased peripheral and CSF‐located ‘LTi‐like’ ILC3 in untreated MS patients,^13^, ^14^, ^15^, ^21^ which were restored to healthy levels by daclizumab,^13^, ^21^ while Gross *et al*.^3^ found decreased circulating ‘LTi‐like’ ILC3 at 6 months, but not at 12 months following alemtuzumab treatment. Hence, declining ILC3 may be a short‐term consequence of alemtuzumab induction and thus not displayed by our data. Nevertheless, those comparisons should be interpreted with caution, as different research groups define ILC according to distinct criteria and markers.^9^

Apart from reported ILC alterations associated with daclizumab and alemtuzumab treatment, Eken *et al*.^22^ investigated helper ILC subsets in a fingolimod‐treated MS patient cohort. In this study, fingolimod caused a generalised ILC‐penia with reduced cell counts in ILC1, ILC2 andILC3. However, the ILC1 compartment was affected less and remained stable in contrast to ILC2 and ILC3, when analysed as a proportion within the helper ILC compartment.^22^ This prompted us to also investigate ILC proportions among helper ILC (in addition to total cell counts and as proportions of PBMC), resulting in our novel observations of marked MS‐related shifts from ILC1 to ILC2 within the helper ILC compartment. These changes occurred independently of alemtuzumab treatment.

To date, little is known about the role of helper ILC1 and ILC2 in MS pathogenesis. One study found ILC1 to be increased in untreated MS patients among total ILC (including NK cells),^14^ while there are no reports on peripheral ILC within the helper ILC compartment in MS patients except for fingolimod‐treated patients as mentioned above.^22^ Experimental autoimmune encephalomyelitis (EAE) models demonstrate ILC1 and/or ILC3‐mediated T cell regulation,^18^, ^23^ while controversial results have been described for ILC2. While deficient ILC2 may increase susceptibility to EAE, treatment with IL‐33 (stimulating ILC2) may impede EAE in an SJL mouse model.^24^ Contrastingly, ILC2 may drive CNS demyelination in an HSV‐IL‐2 murine model, implicating negative impacts of ILC2 in MS.^25^

Considering our observed MS‐related shifts from ILC1 to ILC2, we hypothesised alterations in ILC migratory behaviour as a potential cause. Strikingly, we found abated circulating CCR6^+^ helper ILC1 and ILC2 proportions in MS patients compared with control subjects, which remained changed upon alemtuzumab treatment. However, when CCR6^+^ ILC1 and ILC2 were analysed as percentage of total helper ILC, CCR6^+^ ILC1, but not CCR6^+^ ILC2 or ILC3, were reduced. CCR6‐expressing immune cells are attracted to sites of inflammation by their ligand CCL20.^17^ Alterations in CCR6^+^ ILC have also been reported in psoriatic arthritis, with diminished CCR6^+^ ILC in peripheral blood but enhanced numbers in synovial fluid.^26^ Furthermore, Grigg *et al*.^18^ have recently used an EAE model to identify CCR6‐expressing ILC3 that activate myelin‐specific T cells via MHCII upon CNS migration. The proportional decreases in circulating CCR6^+^ ILC1 and ILC2 therefore suggest MS‐related migration of CCR6^+^ ILC to the CNS, influencing local T cell regulation.

Unfortunately, limits to sample collection did not allow start‐to‐finish follow‐ups among our study participants. Furthermore, only three MS patients relapsed throughout the study. Hence, future studies with larger relapsing patient cohorts are required to address distinct CD56^bright^ NK cell alterations in response to immediate versus chronic disease activity. Although alemtuzumab treatment did not show long‐term impacts on reversing MS‐related shifts among ILC, short‐term effects would be of explicit interest. Moreover, albeit beyond the scope of this study, *in vitro* studies on ILC migratory behaviour in MS are required to elucidate the identified shifts among certain ILC immunophenotypes.

In conclusion, this study elucidates MS‐ and alemtuzumab‐induced dynamics within the circulating ILC population. Intriguingly, we found significant alterations in patients *prior* to alemtuzumab, but no apparent long‐term effects on ILC following alemtuzumab. Furthermore, in those few patients who relapsed long after alemtuzumab treatment, striking alterations in ILC numbers and proportions were seen as MS became active, suggesting that ILC dysregulation may be one of the first indications of the resumption of autoimmunity. Of course, it would be interesting to now explore the function of the ILC subpopulations, especially regarding the role of CCR6^+^ helper ILC and their potential migratory capacities to the CNS. Finally, in the future, we advocate for uniform gating strategies to identify helper ILC populations and focus on shifts within the helper ILC compartment, as helper ILC are proportionately rare populations within blood and tissues.

## Methods

### Study participants

Ethical consent for the study was obtained from the Research Integrity and Ethics Administration of the University of Sydney (project numbers 2018/708 and 2018/377). This study was performed according to the Declaration of Helsinki. Written informed consent was obtained from all participants. MS was defined by McDonald 2017 criteria.^27^ No patients were on DMT at the time of initial blood sampling. A total of 23 MS patients were included in this study. MS patient blood samples taken prior to alemtuzumab treatment (*prior*, *n* = 11) were treatment naïve (6/11) or free from treatment for at least 1 month prior to alemtuzumab (5/11). All patients had low disability (EDSS range 0–2.5). MS activity was defined as new T2 and/or T1 Gadolinium‐enhancing lesions in the 6 months prior to starting alemtuzumab (18/23: active MS, 5/23: inactive MS). Patient data are shown in Supplementary tables 2 and 3.

The first course of alemtuzumab was given for five consecutive days, with a repeated second course 12 months later over 3 days. Blood samples were taken from three groups of MS patients after alemtuzumab. *Post1* was taken within 12 months of alemtuzumab (3/9 after first dose, 6/9 after second). One patient did not have PBMC counted, so 8/9 patients were included for cell counts. *Post2* was taken 29–39 months after first treatment (*n* = 10). The relapse cohort (*n* = 3) were patients that relapsed (confirmed by MRI) after two courses of alemtuzumab treatment. Here, blood samples were taken within 1 month of symptoms and prior to further corticosteroid or alemtuzumab treatment. Age‐ and sex‐matched non‐MS control subjects (*n* = 9) were included in the study, with samples taken from a single timepoint.

### Blood sampling

Peripheral blood mononuclear cells were isolated from blood and collected in EDTA vacuette tubes (Greiner Bio‐One International, Kremsmünster, Austria) using a Ficoll‐Paque Plus (GE Healthcare, Chicago, Illinois, US) density separation gradient. Samples were cryopreserved in 5% dimethyl sulfoxide/foetal bovine serum for storage in liquid nitrogen prior to mass cytometry staining.

### Analysis by mass cytometry and cell staining

Conventionally, flow cytometry is used for the detection of ILC. ILC are regarded as lineage‐negative (Lin^−^) cells. To identify ILC, other peripheral immune cell types are usually excluded with the use of lineage cocktails. However, these have not been standardised to date, making comparisons of currently published results problematic.^9^ Instead of conventional flow cytometry, here we use mass cytometry (cytometry by time‐of‐flight, CyTOF). This novel technology combines flow cytometry and elemental mass spectrometry.^28^ Heavy metal‐isotope antibodies are applied instead of fluorochromes, which prevents spectral overlap and thereby allows more than 40 different antibodies per panel, facilitating comprehensive immunophenotyping,^28^, ^29^ while concomitantly single‐colour lineage cocktails become redundant.

For the CyTOF analysis, cells were prepared and analysed as previously published by our research group^30^: 2.5 x 10^6^ cells were thawed in a 37°C waterbath and washed in Roswell Park Memorial Institute medium. Individual patient/timepoint samples were first barcoded with anti‐human CD45 (on 4 different metal isotopes; BioLegend, San Diego, CA, USA) and blocked with purified human FcR binding inhibitor (eBioscience Inc., San Diego, CA, USA) for 30 min, such that four independent samples could be combined for further staining as described.^31^ To control for batch variability, PBMC taken from a single non‐MS control (taken from a single timepoint) were included within each batch as an internal control and used across 25 batches. Samples were combined (for 7.5–10 x 10^6^ cells) and stained with cisplatin (Fluidigm, South San Francisco, CA, USA) for 5 min as a live/dead marker. Cells were stained with antibodies specific for the markers in Supplementary table 1 for 30 min. These antibodies were purchased unlabelled in a carrier‐protein‐free and conjugated with the indicated metal isotope using the ×8 MaxPAR conjugation kit (Fluidigm) according to the manufacturer's protocol. Conjugations done by the Ramaciotti Facility for Human Systems Biology are indicated in Supplementary table 1. Cells were fixed in 4% paraformaldehyde (PFA) for 20 min prior to being incubated in Foxp3 permeabilisation buffer (eBioscience Inc.) for 15 min. Cells were then stained with an intracellular antibody cocktail (indicated in Supplementary table 1) for 45 min at room temperature (20–24°C). Cells were finally resuspended in DNA intercalator mix (1/1000 iridium intercalator (Fluidigm) in 4% PFA) and left in the fridge until running on a CyTOF 2 Helios mass cytometer (Fluidigm) within 7 days. Cells were washed in Maxpar® Cell Acquisition Solution (CAS) (Fluidigm) before being resuspended in 10% EQ four element beads (Fluidigm) in CAS at a concentration of 0.8 x 10^6^ cells mL^−1^.

### Data analysis

Samples were initially gated using FlowJo v10.8.0 (Becton Dickinson, Ashland, OR, USA) to identify CD45^+^ live single cells (Supplementary figure 4). Among CD45^+^ PBMC, ILC were then defined as CD45^+^ PBMC, negative for lineage markers (Lin^−^) CD3, CD19, CD14, CD11c, CD123, CD34, FcεRIα and TCRab. Lineage‐positive cells were eliminated by biaxial gating as shown in Figure 1. Lin^−^ cells were then sorted into Lin^−^CD56^+^CD94^+^ NK cells (and then further categorised into CD56^bright^ and CD56^dim^ NK cells) and Lin^−^CD56^−^CD94^−^CD127^+^ helper ILC (which were then subdivided into CD294^−^CD117^−^ ILC1, CD294^+^CD117^+/−^ ILC2 and CD294^−^CD117^+^ ILC3) (Figure 1).

Dimensionality reduction was done on all Lin^−^CD127^+^ helper ILC (from all patients) using ‘FIt‐SNE’,^32^ as part of the ‘Spectre’ package in R.^33^ Dimensionality reduction plots were calculated using the following markers: CD1a, CD1c, CD5, CD21, CD23, CD38, CD80, CD86, CD103, CD117 (c‐kit), CD120a, CD120b, CD161, CD184 (CXCR4), CD196 (CCR6), CD213α1, CD213α2, CD274 (PD‐L1), CD294 (CRTH2), CD335 (NKp46), CD336 (NKp44), GATA3, PAF‐R (platelet‐activating factor receptor) and RORγt, T‐bet (Supplementary table 1).

### Statistical analysis

All statistics were calculated using packages available within R,^34^ using Type III Sum of Squares.^30^

To calculate differences between groups, a permutational multivariate analysis of variance (PERMANOVA) was done using the package ‘vegan’.^35^ 4999 permutations were done to generate *P*‐values, to provide power and confidence for α = 0.01.^36^ For pairwise comparisons, the package ‘pairwiseAdonis’ was used with Holm's correction for multiple comparisons.^37^

For comparisons of ILC subset levels (either proportions or cell counts) between all five groups (*non‐MS*, *prior*, *post1*, *post2* and *relapse*), a PERMANOVA was done followed by pairwise comparisons with Holm's correction as discussed above. *Prior*, *post2* and *relapse* groups were compared to *non‐MS* controls (for three comparisons). When comparing between MS patients before and after treatment, a linear mixed‐effects model was calculated using the ‘lme4’ package.^38^ Individual patients were considered random effects for a repeated measures test that accommodated missing values, as not all patients had all available timepoints, while timepoints (*prior*, *post1* and *post2*) were fixed effects. 4999 permutations were then run using the ‘permanova.lmer’ function as part of the ‘predictmeans’ R package to calculate *P*‐values.^39^, ^40^ Five multiple comparisons were made: *prior* to *post1*, *post2* and *relapse*; *post1* to *post2*; and *post2* to *relapse*. The functions ‘permmodels’ and ‘predictmeans’ (also part of the ‘predictmeans’ package) generated 4999 permutations to calculate *P*‐values with Holm's correction. *P*‐values <0.05 were considered statistically significant. All plots were generated using GraphPad Prism software (GraphPad Software, San Diego, CA, USA) version 9.4.1.

## Author Contributions

**Florentina Aglas‐Leitner:** Conceptualization; formal analysis; investigation; visualization; writing – original draft; writing – review and editing. **Pierre Juillard:** Data curation; methodology; writing – review and editing. **Annette Juillard:** Data curation; methodology; writing – review and editing. **Scott N Byrne:** Conceptualization; investigation; project administration; supervision. **Simon Hawke:** Conceptualization; funding acquisition; project administration; supervision; writing – review and editing. **Georges E Grau:** Conceptualization; funding acquisition; investigation; project administration; supervision; writing – review and editing. **Felix Marsh‐Wakefield:** Conceptualization; data curation; formal analysis; investigation; methodology; software; supervision; visualization; writing – review and editing.

## Conflict of interest

Dr Hawke has received research funding, travel grants and honoraria from Sanofi. All other authors have no conflict of interest to report.

## Supporting information


Supporting Information

## References

[1] Rommer PS , Milo R , Han MH *et al*. Immunological aspects of approved MS therapeutics. Front Immunol 2019; 10: 1564. 31354720 10.3389/fimmu.2019.01564 PMC6637731

[2] Lunemann JD , Ruck T , Muraro PA , Bar‐Or A , Wiendl H . Immune reconstitution therapies: concepts for durable remission in multiple sclerosis. Nat Rev Neurol 2020; 16: 56–62. 31649335 10.1038/s41582-019-0268-z

[3] Gross CC , Ahmetspahic D , Ruck T *et al*. Alemtuzumab treatment alters circulating innate immune cells in multiple sclerosis. Neurol Neuroimmunol Neuroinflamm 2016; 3: e289. 27766281 10.1212/NXI.0000000000000289 PMC5063395

[4] Evan JR , Bozkurt SB , Thomas NC , Bagnato F . Alemtuzumab for the treatment of multiple sclerosis. Expert Opin Biol Ther 2018; 18: 323–334. 29309202 10.1080/14712598.2018.1425388

[5] Hemmer B , Kerschensteiner M , Korn T . Role of the innate and adaptive immune responses in the course of multiple sclerosis. Lancet Neurol 2015; 14: 406–419. 25792099 10.1016/S1474-4422(14)70305-9

[6] Gandhi R , Laroni A , Weiner HL . Role of the innate immune system in the pathogenesis of multiple sclerosis. J Neuroimmunol 2010; 221: 7–14. 19931190 10.1016/j.jneuroim.2009.10.015 PMC2854189

[7] Vivier E , Artis D , Colonna M *et al*. Innate lymphoid cells: 10 years on. Cell 2018; 174: 1054–1066. 30142344 10.1016/j.cell.2018.07.017

[8] Spits H , Artis D , Colonna M *et al*. Innate lymphoid cells — a proposal for uniform nomenclature. Nat Rev Immunol 2013; 13: 145–149. 23348417 10.1038/nri3365

[9] Trabanelli S , Gomez‐Cadena A , Salome B *et al*. Human innate lymphoid cells (ILCs): toward a uniform immune‐phenotyping. Cytometry B Clin Cytom 2018; 94: 392–399. 29244250 10.1002/cyto.b.21614

[10] Melo‐Gonzalez F , Hepworth MR . Functional and phenotypic heterogeneity of group 3 innate lymphoid cells. Immunology 2017; 150: 265–275. 27935637 10.1111/imm.12697 PMC5290240

[11] Yang Y , Day J , Souza‐Fonseca Guimaraes F , Wicks IP , Louis C . Natural killer cells in inflammatory autoimmune diseases. Clin Transl Immunology 2021; 10: e1250. 33552511 10.1002/cti2.1250 PMC7850912

[12] Xiong T , Turner JE . Innate lymphoid cells in autoimmunity and chronic inflammatory diseases. Semin Immunopathol 2018; 40: 393–406. 29568972 10.1007/s00281-018-0670-4

[13] Perry JS , Han S , Xu Q *et al*. Inhibition of LTi cell development by CD25 blockade is associated with decreased intrathecal inflammation in multiple sclerosis. Sci Transl Med 2012; 4: 145ra106. 10.1126/scitranslmed.3004140 PMC3846177 22855463

[14] Gross CC , Schulte‐Mecklenbeck A , Hanning U *et al*. Distinct pattern of lesion distribution in multiple sclerosis is associated with different circulating T‐helper and helper‐like innate lymphoid cell subsets. Mult Scler 2017; 23: 1025–1030. 27481205 10.1177/1352458516662726

[15] Degn M , Modvig S , Dyring‐Andersen B *et al*. Increased prevalence of lymphoid tissue inducer cells in the cerebrospinal fluid of patients with early multiple sclerosis. Mult Scler 2016; 22: 1013–1020. 26453677 10.1177/1352458515609795

[16] Laroni A , Uccelli A . CD56bright natural killer cells: a possible biomarker of different treatments in multiple sclerosis. J Clin Med 2020; 9: 1450. 32414131 10.3390/jcm9051450 PMC7291063

[17] Meitei HT , Jadhav N , Lal G . CCR6‐CCL20 axis as a therapeutic target for autoimmune diseases. Autoimmun Rev 2021; 20: 102846. 33971346 10.1016/j.autrev.2021.102846

[18] Grigg JB , Shanmugavadivu A , Regen T *et al*. Antigen‐presenting innate lymphoid cells orchestrate neuroinflammation. Nature 2021; 600: 707–712. 34853467 10.1038/s41586-021-04136-4 PMC8702489

[19] Gilmore W , Lund BT , Li P *et al*. Repopulation of T, B, and NK cells following alemtuzumab treatment in relapsing‐remitting multiple sclerosis. J Neuroinflammation 2020; 17: 189. 32539719 10.1186/s12974-020-01847-9 PMC7296935

[20] Gross CC , Schulte‐Mecklenbeck A , Runzi A *et al*. Impaired NK‐mediated regulation of T‐cell activity in multiple sclerosis is reconstituted by IL‐2 receptor modulation. Proc Natl Acad Sci USA 2016; 113: E2973–E2982. 27162345 10.1073/pnas.1524924113 PMC4889377

[21] Lin YC , Winokur P , Blake A , Wu T , Romm E , Bielekova B . Daclizumab reverses intrathecal immune cell abnormalities in multiple sclerosis. Ann Clin Transl Neurol 2015; 2: 445–455. 26000318 10.1002/acn3.181 PMC4435700

[22] Eken A , Yetkin MF , Vural A *et al*. Fingolimod alters tissue distribution and cytokine production of human and murine innate lymphoid cells. Front Immunol 2019; 10: 217. 30828332 10.3389/fimmu.2019.00217 PMC6385997

[23] Brown MA , Russi AE . (T)Betting on innate lymphoid cells in CNS inflammatory disease. Nat Immunol 2017; 18: 1063–1064. 28926546 10.1038/ni.3839

[24] Brown MA , Weinberg RB . Mast cells and innate lymphoid cells: underappreciated players in CNS autoimmune demyelinating disease. Front Immunol 2018; 9: 514. 29619025 10.3389/fimmu.2018.00514 PMC5871669

[25] Hirose S , Jahani PS , Wang S *et al*. Type 2 innate lymphoid cells induce CNS demyelination in an HSV‐IL‐2 mouse model of multiple sclerosis. iScience 2020; 23: 101549. 33083718 10.1016/j.isci.2020.101549 PMC7522755

[26] Leijten EF , van Kempen TS , Boes M *et al*. Enrichment of activated group 3 innate lymphoid cells in psoriatic arthritis synovial fluid. Arthritis Rheumatol 2015; 67: 2673–2678. 26137857 10.1002/art.39261

[27] Thompson AJ , Banwell BL , Barkhof F *et al*. Diagnosis of multiple sclerosis: 2017 revisions of the McDonald criteria. Lancet Neurol 2018; 17: 162–173. 29275977 10.1016/S1474-4422(17)30470-2

[28] Spitzer MH , Nolan GP . Mass cytometry: single cells, many features. Cell 2016; 165: 780–791. 27153492 10.1016/j.cell.2016.04.019 PMC4860251

[29] Hartmann FJ , Bendall SC . Immune monitoring using mass cytometry and related high‐dimensional imaging approaches. Nat Rev Rheumatol 2020; 16: 87–99. 31892734 10.1038/s41584-019-0338-z PMC7232872

[30] Marsh‐Wakefield F , Juillard P , Ashhurst TM *et al*. Peripheral B‐cell dysregulation is associated with relapse after long‐term quiescence in multiple sclerosis patients. Immunol Cell Biol 2022; 100: 453–467. 35416319 10.1111/imcb.12552 PMC9322415

[31] Wagar LE . Live cell barcoding for efficient analysis of small samples by mass cytometry. Methods Mol Biol 2019; 1989: 125–135. 31077103 10.1007/978-1-4939-9454-0_9

[32] Linderman GC , Rachh M , Hoskins JG , Steinerberger S , Kluger Y . Fast interpolation‐based t‐SNE for improved visualization of single‐cell RNA‐seq data. Nat Methods 2019; 16: 243–245. 30742040 10.1038/s41592-018-0308-4 PMC6402590

[33] Ashhurst TM , Marsh‐Wakefield F , Putri GH *et al*. Integration, exploration, and analysis of high‐dimensional single‐cell cytometry data using Spectre. Cytometry A 2022; 101: 237–253. 33840138 10.1002/cyto.a.24350

[34] R Core Team . R: A Language and Environment for Statistical Computing. Vienna: R Foundation for Statistical Computing; 2019.

[35] Oksanen J , Blanchet FG , Friendly M , *et al*. vegan: community ecology package. R package version 2.5–7; 2020.

[36] Manly BFJ . Randomization, Bootstrap and Monte Carlo methods in Biology. 2nd ed. London: Chapman & Hall; 1997.

[37] Arbizu PM . pairwiseAdonis: pairwise multilevel comparison using adonis. R package version 0.4.; 2017.

[38] Bates D , Mächler M , Bolker B , Walker S . Fitting linear mixed‐effects models using lme4. J Stat Softw 2015; 67: 1–48.

[39] Lee OE , Braun TM . Permutation tests for random effects in linear mixed models. Biometrics 2012; 68: 486–493. 21950470 10.1111/j.1541-0420.2011.01675.x PMC3883440

[40] Luo D , Ganesh S , Koolaard J . predictmeans: Calculate predicted means for linear models. R package version 1.0.6.; 2021.

