# Supplementary material for: Circulating CCR6 + ILC proportions are lower in multiple sclerosis patients
Source: Clin Transl Immunology. 2022 Dec 23;11(12):e1426. doi: 10.1002/cti2.1426 (PMC9782758; doi:10.1002/cti2.1426)
Supplement: Supplementary file 1 — Supporting Information [file CTI2-11-e1426-s001.pdf]

Supplementary figure 1

(a)

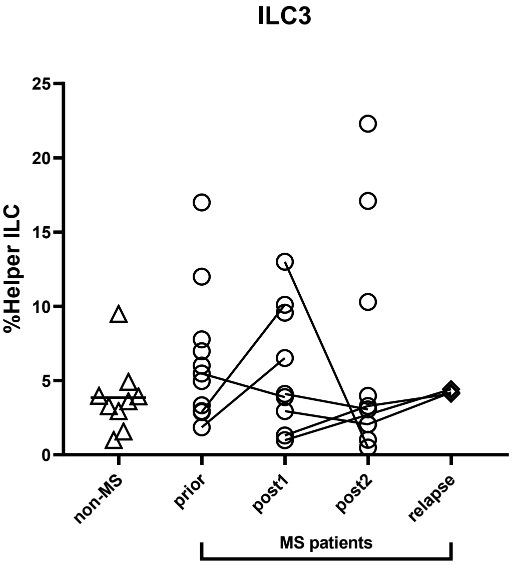

Supplementary figure 1: ILC3 subset shifts within the Lin$^-$CD127$^+$ helper ILC compartment in MS patients.

(**a**) Levels of ILC3 (CD294$^-$CD117$^+$) as proportions of helper ILC across timepoints (%Helper ILC). Helper ILC were defined as live CD45$^+$Lin$^-$CD127$^+$ (CD3$^-$CD19$^-$CD14$^-$CD11c$^-$CD123$^-$CD34$^-$FcεRIα$^-$TCRab$^-$) CD56$^-$CD94$^-$. For comparisons of cell percentages across timelines (*non-MS* (multiple sclerosis) controls (n = 9), untreated MS patients (*prior*, n = 11), and MS patients *post-1* (< 12 months after alemtuzumab dose, n = 9), *post-2* (> 12 months, n = 10) alemtuzumab and *relapse* (n = 3)), a PERMANOVA was done followed by pairwise comparisons with Holm's correction. *Prior*, *post-2* and *relapse* groups were compared to *non-MS* controls (for three comparisons). Five multiple comparisons were made (*prior* to *post-1*, *post-2* and *relapse*, and *post*-1 to *post-2*, and *post-2* to *relapse*) using a further 4999 permutations with Holm's correction. 4999 permutations were run to calculate *P*-values. Mean is shown across groups, *P*-values < 0.1 are shown.

Supplementary figure 2

(a) **(b)**

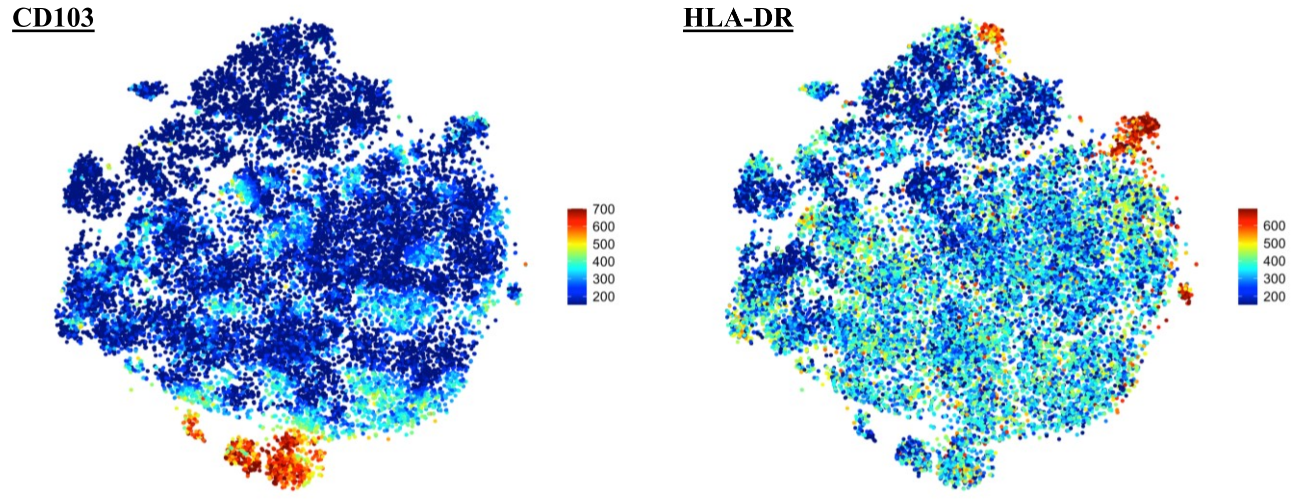

Supplementary figure 2: CD103$^+$ and HLA-DR$^+$ Helper ILC Immunophenotypes

FIt-SNE plots generated on helper ILC (live CD45$^+$Lin$^-$CD127$^+$ (CD3$^-$CD19$^-$CD14$^-$CD11c$^-$CD123$^-$CD34$^-$FcεRIα$^-$TCRab$^-$CD56$^-$CD94$^-$)). Dimensionality reduction was done on all Lin$^-$CD127$^+$ helper ILC (from all patients). Dimensionality reduction plots were calculated using markers in Supplementary table 1. Identification of rare (**a**) CD103$^{+/-}$, and (**b**) HLA-DR$^+$ helper ILC immunophenotypes.

Supplementary figure 3

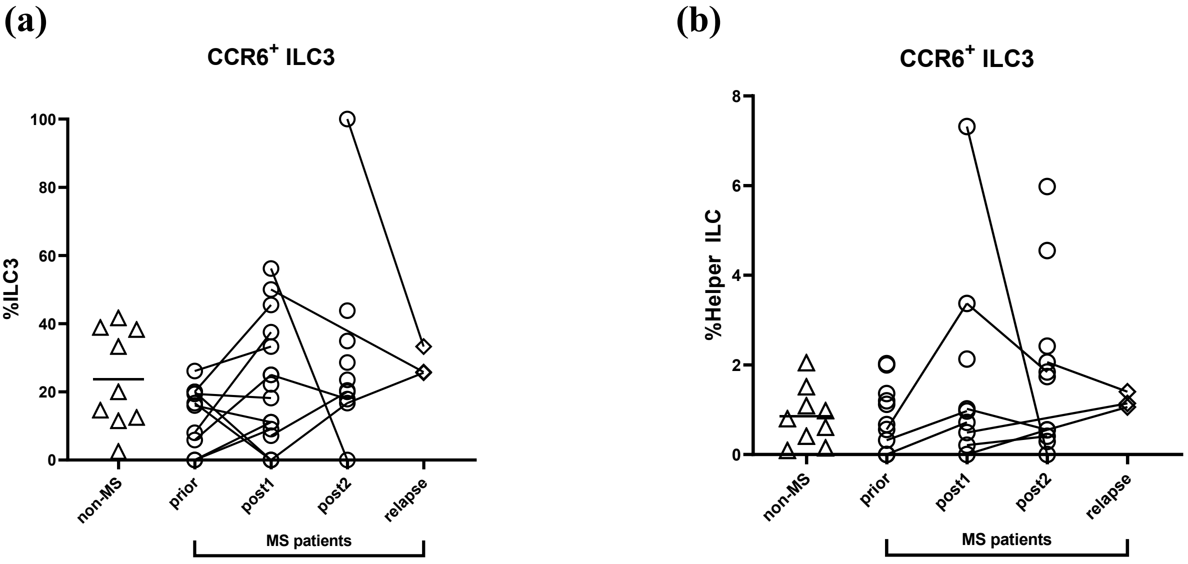

Supplementary figure 3: Stable CCR6$^+$ ILC3 in MS patients.

Comparisons of CCR6+ ILC3 as proportions of (**a**) ILC3 (%ILC3) and (**b**) helper ILC (%Helper ILC) between all five groups (*non-MS* controls (n = 9), untreated MS patients (*prior*, n = 10), and MS patients *post-1* (< 12 months after alemtuzumab dose, n = 8/9), *post-2* (> 12 months, n = 10) alemtuzumab and *relapse* (n = 3)), a PERMANOVA was done followed by pairwise comparisons with Holm's correction. *Prior*, *post-2* and *relapse* groups were compared to *non-MS* controls (for three comparisons). A linear mixed-effects model was calculated when comparing between MS patients before and after treatment. 4999 permutations were then run to calculate *P*-values. Five multiple comparisons were made (*prior* to *post-1*, *post-2* and *relapse*, and *post*-1 to *post-2*, and *post-2* to *relapse*) using a further 4999 permutations with Holm's correction. Mean is shown in *non-MS* controls, *P*-values < 0.1 are shown.

Supplementary figure 4

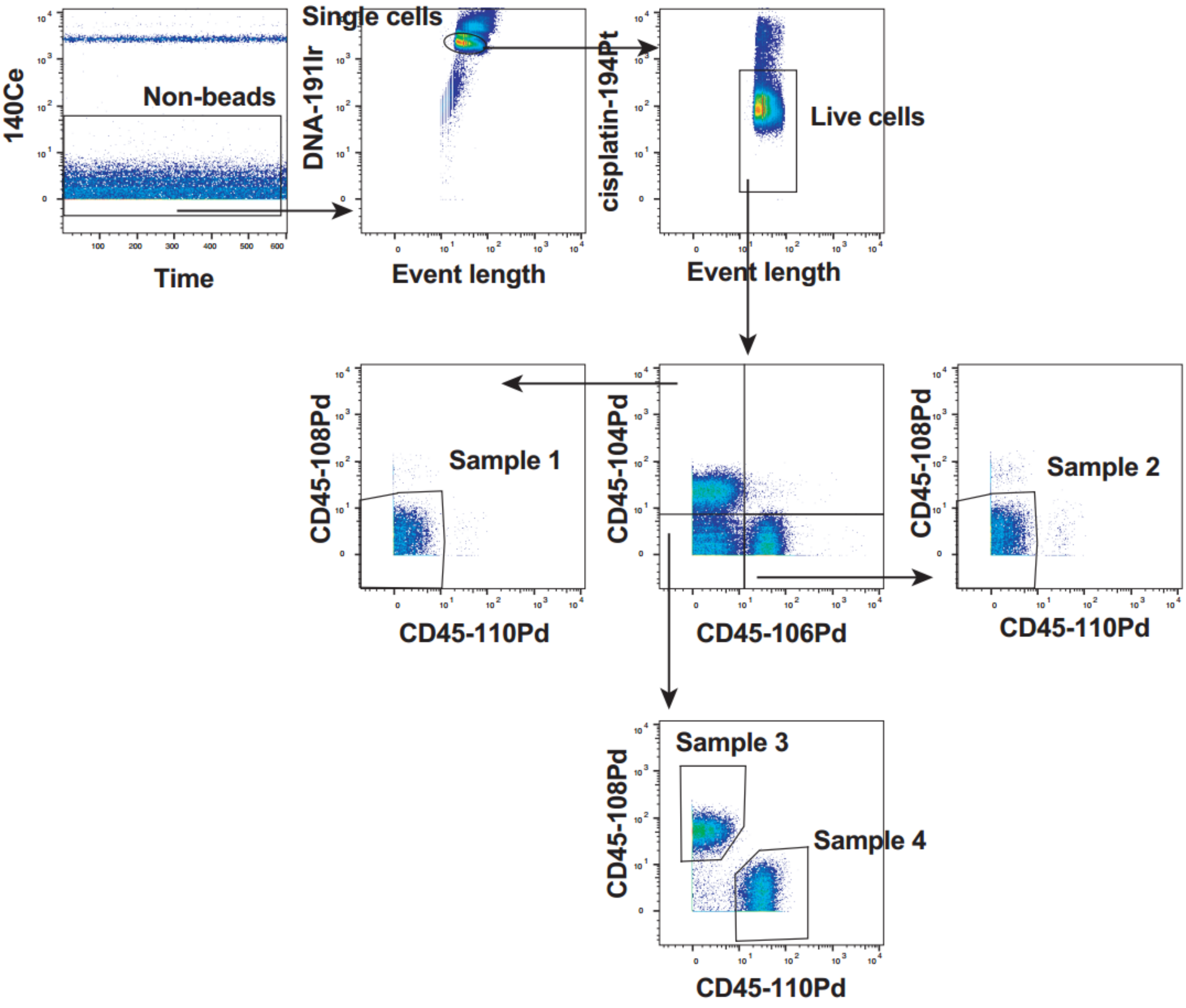

<u>**Supplementary figure 4: Gating strategy for live CD45$^+$ PBMC**</u>

Of all acquired events, beads were first excluded with the use of 140Ce isotope. Cell aggregates were then removed by gating 191Ir DNA signal (from staining with an Ir-loaded DNA calculator) versus event length. Live cells were identified based on cisplatin. CD45$^+$ PBMC were subsequently identified by barcoding with 4 different isotopes (104Pd, 106Pd, 108Pd, 110Pd).

Supplementary table 1: 43-parameter CyTOF panel for ILC analysis

TARGET	ISOTOPE	CLONE	COMPANY	FIt-SNE plots
CD1a	167Er§	HI149	BioLegend	✓
CD1c	163Dy§	L161	BioLegend	✓
CD3	115In	UCHT1	BioLegend	
CD4	168Er§	SK3	BioLegend	
5-HT2A		9B11.1	Merck KGaA (Darmstadt, Germany)	
CD5	148Nd	UCHT2	BioLegend	✓
CD8a	89Y§	RPA-T8	BD Biosciences (Franklin Lakes, NJ, USA)	
CD11c	172Yb	Bu15	BioLegend	
CD14	160Gd§	M5E2	BD Biosciences	
CD19	142Nd§	HIB19	BioLegend	
CD21	152Sm§	BU32	BioLegend	✓
CD23	169Tm§	EBVCS-5	BioLegend	✓
CD34	166Er§	581	BD Biosciences	
CD38	145Nd	HIT2	BioLegend	✓
CD45†	104Pd§<br>106Pd§<br>108Pd§<br>110Pd§	HI30	BioLegend	
CD56	113In§	NCAM16.2	BD Biosciences	
CD80	162Er§	L307.4	BD Biosciences	✓
CD86	156Gd§	IT2.2	BD Biosciences	✓
CD94	141Pr§	DX22	BioLegend	
CD103	155Gd	Ber-ACT8	BioLegend	✓
CD117	143Nd§	104D2	BioLegend	✓
CD120a	158Gd	REA252	Miltenyi Biotec (Bergisch Gladbach, Germany)	✓
CD120b	159Tb	3G7A02	BioLegend	✓
CD123	151Eu§	6H6	BioLegend	
CD127	165Ho§	A019D5	BioLegend	
CD161 (KLRB1)	164Er§	DX12	BD Biosciences	✓
CD184 (CXCR4)	175Lu§	12G5	BD Biosciences	✓
CD196 (CCR6)	154Sm§	REA190	Miltenyi Biotec	✓
CD213a1	170Er	SS12B	BioLegend	✓
CD213a2	176Yb	REA308	Miltenyi Biotec	✓
CD274 (PD-L1)	161Dy§	29E.2A3	BioLegend	✓
CD294 (CRTH2)	153Eu	BM16	BioLegend	✓
CD335 (NKp46)	171Yb§	9E2 NKp46	BioLegend	✓
CD336 (NKp44)	149Sm	P44-8	BioLegend	✓
FceR1	146Nd§	AER-37 (CRA-1)	BioLegend	
TCRab		T10B9.1A-31		
GATA3 ‡	139La	TWAJ	eBioscience Inc.	✓
HLA-DR	174Yb§	L243	BioLegend	
PAF-R	150Nd	11A4, Clone 21	Cayman Chemicals (Ann Arbor, Michigan, USA)	✓
RORgt ‡	147Sm§	Primary antibody: AF647 conjugate, clone Q21-559. Secondary antibody: anti-Cy5, clone CY5-15.	RORgt from BD Biosciences; anti-Cy5 from Sigma (St. Louis, Missouri, US)	✓
Tbet ‡	209Bi§	4B10	BD Biosciences	✓
TCRgd	144Nd§	B1	BioLegend	
TCR Va 7.2	173Yb§	3C10	BioLegend	

§Isotopes conjugated by the Ramaciotti Facility for Human Systems Biology, The University of Sydney, Australia.
†CD45 was used for barcoding to allow up to three samples to be stained together. ‡Markers stained intracellularly

Supplementary table 2: Study participant age and sex.

PATIENT ID	SEX	AGE§	DISEASE DURATION (YEARS)§
MS patients	*74% F*	*Median = 40.2*	*Median = 6.0*
**MS01**	F	38.8	6.1
**MS02**	F	36	9.2
**MS03**	M	43.1	23.6
**MS04**	F	25.1	3.3
**MS05**	F	34.7	11.9
**MS06**	F	52	24.8
**MS07**	F	40.2	7.7
**MS08**	M	33.1	0.3
**MS09**	F	46.9	4.3
**MS10**	F	32	2.8
**MS11**	M	46.1	19.3
**MS12**	M	53.4	0.1
**MS13**	F	40.2	3.3
**MS14**	M	46.4	8.8
**MS15**	F	40.2	6.0
**MS16**	F	25.2	0.4
**MS17**	F	37.2	17.7
**MS18**	F	40.2	5.9
**MS19**	M	36.7	0.4
**MS20**	F	35.8	6.3
**MS21**	F	37.4	2.4
**MS22**	F	44.8	0.2
**MS23**	F	45.5	18.6
Non-MS patients	*78%F*	*Median = 39.0*	
**Non-MS01**	F	40.8	
**Non-MS02**	F	36.8	
**Non-MS03**	M	41.4	
**Non-MS04**	F	23.5	
**Non-MS05**	F	33.1	
**Non-MS06**	F	54.9	
**Non-MS07**	F	39	
**Non-MS08**	F	27.5	
**Non-MS09**	M	42.3	

§at first timepoint in which the participant had blood taken in the study – for further information, see Supplementary table 3.

Supplementary table 3: Treatment regimen and disease activity of MS participants.

PATIENT ID	MONTHS SINCE FIRST DOSE OF ALEMTUZUMAB				PREVIOUS DMT	MONTHS SINCE LAST TREATMENT PRIOR TO ALEMTUZUMAB	ACTIVE MS§
	Prior	*Post1*	*Post2*	*Relapse*			
**MS PATIENTS** *n = 23*	*n = 11*	*n = 9*	*n = 10*	*n = 3*	*previous DMT: n = 15/23 Treatment-naïve: n = 8/23*	*Median = 3 months Range = 1-15 months*	*Active: n = 18/23 Non-active: n = 5/23*
**MS01**		24	32		Fingolimod	2	Yes
**MS02**		21	36	39	Azathioprine + IVIG	1	Yes
**MS03**		19		34	Natalizumab	1	No
**MS04**			31		—/—	—/—	Yes
**MS05**			39		Fingolimod	4	Yes
**MS06**			30	41	Natalizumab	1	No
**MS07**		11†	36		Dimethyl fumarate	3	Yes
**MS08**	X	18			—/—	—/—	Yes
**MS09**		22	38		Fingolimod	2	Yes
**MS10**	X	11			Fingolimod	6	Yes
**MS11**			39		—/—	—/—	Yes
**MS12**	X	20			—/—	—/—	Yes
**MS13**			32		Fingolimod	3	Yes (+ clinical)
**MS14**		8			Fingolimod	3	Yes
**MS15**			29		Fingolimod	3	No (but clinical)
**MS16**	X‡				Dimethyl fumarate	2	Yes
**MS17**	X				—/—	—/—	Yes
**MS18**	X				Fingolimod	15	Yes
**MS19**	X				—/—	—/—	Yes
**MS20**	X				—/—	—/—	Yes
**MS21**	X				Fingolimod	2	No
**MS22**	X				—/—	—/—	Yes
**MS23**	X				Fingolimod	15	No

§Defined as new T2 and/or T1 Gad-enhancing lesions in the 6 months prior to starting alemtuzumab

†No PBMC count taken for this sample

‡No CCR6 staining for this sample, so was excluded from all CCR6 analyses
